# P-1812. Understanding the role of the non-coding control region in JC polyomavirus cell tropism and central nervous system entry

**DOI:** 10.1093/ofid/ofaf695.1981

**Published:** 2026-01-11

**Authors:** Elizabeth Wagstaff, C Sabrain Tan

**Affiliations:** University of Iowa, Iowa City, Iowa; University of Iowa, Iowa City, Iowa

## Abstract

**Background:**

JC Polyomavirus (JCPyV) infects 40-90% of the adult population in a benign kidney infection. However, in a subset of immunocompromised individuals it causes a rapid and often fatal infection known as Progressive Multifocal Leukoencephalopathy (PML). Currently there are no treatments for PML and those that survive the initial infection are left with severe lifelong complications. Much of the process the virus must undergo to travel to the brain and establish infection is currently unknown. The non-coding control region (NCCR) of the viral genome is thought to have an important role in establishing infection in the central nervous system (CNS) as it contains many transcription factor binding sites. In neurotropic strains of the virus there are many insertions, duplications, and deletions in this region that may contribute to cell tropism and increased replication in the CNS. However, two published cases have reported JCPyV in the CNS without NCCR rearrangement suggesting the role of the region may be more complex than previously hypothesized. Studies in this area have been limited previously due to a lack of realistic models for infection and there is confusion over which cells in the CNS are being infected and what changes in the virus permit infection.

**Figure:**
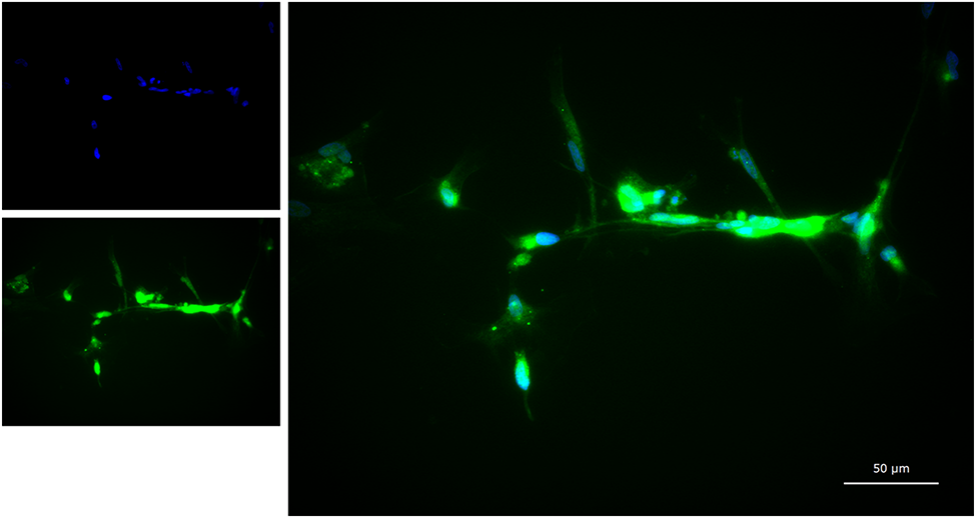
JC virus infection of primary astrocytes RNAscope detection of the human polyomavirus JC virus transcripts in iPSC-derived human astrocytes. Shown is DAPI staining of nuclei (top left and merged images) and detection of viral RNA (bottom left and merged image). Scale bar = 50um

**Figure:**
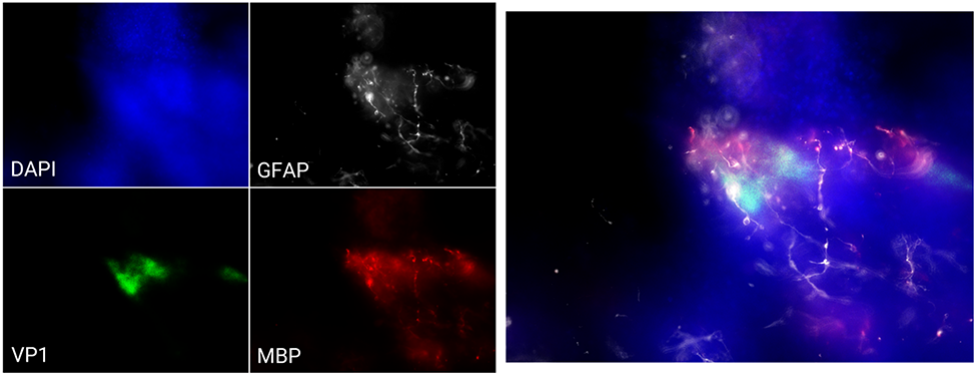
JC virus infection of human brain organoid. JC virus capsid protein VP1 was colocalized with the oligodendrocytes marker MBP, near astrocytes (GFAP). Merged staining on the right include DAPI.

**Methods:**

We have developed several novel primary cell infection models, including a brain organoid model, to test infection by wildtype virus with and without NCCR rearrangements. Additionally, we are using advanced molecular techniques including single cell RNA sequencing to identify the interactions between JCPyV and host cells.

**Results:**

Primary oligodendrocytes and tubule epithelial cells can be infected by wildtype archetype and neurotropic JCPyV. Rearranged JCPyV infects both cell types at a significantly higher level than archetype. Virus with non-rearranged NCCR is capable of infecting oligodendrocytes, but is less efficient than rearranged virus. Brain organoids appear to be infectable with JCPyV neurotropic virus (MAD-1).

**Conclusion:**

The knowledge gained from these studies will aid in understanding how JCPyV changes from a benign kidney disease to a deadly neuro pathogen and potentially lead to innovative therapeutics and preventatives for patients.

**Disclosures:**

All Authors: No reported disclosures

